# Unraveling impact and potential mechanisms of baseline pain on efficacy of immunotherapy in lung cancer patients: a retrospective and bioinformatic analysis

**DOI:** 10.3389/fimmu.2024.1456150

**Published:** 2024-11-25

**Authors:** Zexin Zhang, Wenjie Zhao, Chang Lv, Zexia Wu, Wenhao Liu, Xuesong Chang, Yaya Yu, Zhenzhen Xiao, Yihan He, Haibo Zhang

**Affiliations:** ^1^ The Second Clinical School of Guangzhou University of Chinese Medicine, Guangzhou, China; ^2^ Clinical Medical College of Acupuncture Moxibustion and Rehabilitation, Guangzhou University of Chinese Medicine, Guangzhou, China; ^3^ Deparment of Oncology, Guangdong Province Hospital of Chinese Medicine, Guangzhou, China; ^4^ The Second Affiliated Hospital of Guangzhou University of Chinese Medicine, Guangzhou, China; ^5^ Guangdong Provincial Key Laboratory of Clinical Research on Traditional Chinese Medicine Syndrome, Guangzhou, China; ^6^ Guangdong-Hong Kong-Macau Joint Lab on Chinese Medicine and Immune Disease Research, Guangzhou University of Chinese Medicine, Guangzhou, China; ^7^ State Key Laboratory of Dampness Syndrome of Chinese Medicine, The Second Affiliated Hospital of Guangzhou University of Chinese Medicine, Guangzhou, China

**Keywords:** baseline pain, immunotherapy, lung cancer, prognostic model, peripheral inflammatory cells, C-X-C motif chemokine ligand 12

## Abstract

**Objective:**

Pain is a prevalent discomfort symptom associated with cancer, yet the correlations and potential mechanisms between pain and the efficacy of cancer immunotherapy remain uncertain.

**Methods:**

Non-small cell lung cancer (NSCLC) patients who received immune checkpoint inhibitors (ICIs) in the inpatient department of Guangdong Provincial Hospital of Chinese Medicine from January 1, 2018, to December 31, 2021, were retrospectively enrolled. Through cox regression analysis, prognostic factors and independent prognostic factors affecting the efficacy of ICIs were identified, and a nomogram model was constructed. Hub cancer-related pain genes (CRPGs) were identified through bioinformatic analysis. Finally, the expression levels of hub CRPGs were detected using an enzyme-linked immunosorbent assay (ELISA).

**Results:**

Before PSM, a total of 222 patients were enrolled in this study. Univariate and multivariate cox analysis indicated that bone metastasis and NRS scores were independent prognostic factors for the efficacy of ICIs. After PSM, a total of 94 people were enrolled in this study. Univariate cox analysis and multivariate cox analysis indicated that age, platelets, Dnlr, liver metastasis, bone metastasis, and NRS scores were independent prognostic factors for the efficacy of ICIs. A nomogram was constructed based on 6 independent prognostic factors with AUC values of 0.80 for 1-year, 0.73 for 2-year, and 0.80 for 3-year survival. ELISA assay results indicated that the level of CXCL12 significantly decreased compared to baseline after pain was relieved.

**Conclusion:**

Baseline pain is an independent prognostic factor affecting the efficacy of ICIs in lung cancer, potentially through CXCL12-mediated inflammation promotion and immunosuppression.

## Background

Pain is a prevalent complication of cancer, with an estimated incidence of approximately 44.5% among cancer patients (1). Inadequate management of pain have profound effects on physical and emotional well-being, exacerbating anxiety, anger, and depression, and significantly diminishing quality of life (2–4). Furthermore, pain has been shown to suppress immune responses and facilitate tumor growth (5). Refractory cancer pain significantly diminishes the quality of life for individuals with cancer and is intricately linked to a decline in overall survival (OS) (6). Among patients with lung cancer, as many as 50% will endure pain that detrimentally impacts the efficacy of tumor therapies and their prospects for survival (7).

Tumor immune microenvironment (TME) is a complex component and tumor cells can evade the killing effect of various therapies through reprogramming metabolism (8), angiogenesis and other methods. In recent years, immunotherapy has emerged as a crucial component in the treatment of tumors (9). The effect of this treatment is closely related to the complex composition of the tumor immune infiltrating microenvironment (10, 11). Though some patients may experience long-term survival benefits from ICIs treatment, particularly those in late-stage, the response rate was limited to approximately 23% (12). Furthermore, as high as to 80% patients still exhibit primary drug resistance (13). This resistance persists even in patients with high PD-L1 expression levels, with approximately 50% of these patients showing resistance to ICIs treatment (14). Many studies have indicated that baseline characteristics including age, gender, and brain metastasis can impact the effectiveness of ICIs in lung cancer (15). Nevertheless, there is limited understanding regarding the potential impact of baseline pain on survival outcomes in lung cancer patients undergoing treatment with ICIs.

Certain studies have found that the significance of pain persisted even after accounting for various clinical variables such as age, gender, performance status, and disease stage in multivariable analysis (15, 16). A preliminary investigation into the prognostic value of Patient-Reported Outcomes (PRO) and performance status in forecasting survival among patients with metastatic lung cancer undergoing chemoimmunotherapy indicated that pain levels reported in PRO can be a valuable predictor of OS and progression-free survival (PFS) (17).

Cancer related pain is characterized by a combination of nociceptive and neuropathic components, with evidence suggesting that tumor-infiltrating neutrophils play a role in the development of cancer-related inflammation and neuropathic pain (18, 19). A recent study indicated that neutrophils was demonstrated remarkable complexity, and was characterized by 10 distinct states encompassing inflammation, angiogenesis, and antigen presentation (11). Neutrophil infiltration within the tumor microenvironment (TME) has been shown to potentially elevate peripheral neutrophil levels, which could impede the trafficking of anti-cancer T cells (20). The enumeration of diverse leukocytes and soluble factors in peripheral blood can serve as an indirect indicator of the immune profile of the cancer, with neutrophils and lymphocytes frequently representing the predominant subtypes (21). In accordance with the findings of numerous studies, an elevated baseline neutrophil-to-lymphocyte ratio (NLR) typically correlates with an unfavorable prognosis (22). This adverse association extends to patients undergoing treatment with ICIs. In a comprehensive pan-cancer study, elevated NLR was correlated with decreased OS, PFS, and objective response rate (ORR) (23). Kargl et al. discovered a negative correlation between tumor-infiltrating neutrophils and CD8-expressing T cells in NSCLC specimens (24), with further investigations revealing a connection between intratumoral NLR and reduced effectiveness of ICIs (25). Using a mouse model of lung cancer, the researchers demonstrated that antagonizing neutrophils restored the infiltration of tumor CD8 T cells and improved the efficacy of anti-PD1 treatment (25). In the pathogenesis of nociceptive pain associated with another type of cancer, calcitonin gene-related peptide (CGRP) serves as a crucial signaling molecule that can induce hyperalgesia and impact the tumor microenvironment (26). Cancer cells interact with nociceptor neurons and stimulate the release of CGRP, leading to increased exhaustion of cytotoxic CD8 T cells (27), which in turn hinders their ability to eliminate tumors and diminishes the effectiveness of ICIs (28).

Therefore, we conducted a retrospective analysis to determine whether baseline pain is an independent prognostic factor for ICIs. We also performed a bioinformatics analysis to elucidate the underlying possible mechanisms. Finally, the expression level of hub CRPGs were detected using enzyme linked immunosorbent assay.

## Methods and materials

### Study oversight

In this retrospective cohort study, lung cancer patients who received ICIs treatment were recruited from the inpatient department of Guangdong Provincial Hospital of Chinese Medicine from January 1, 2018, to December 31, 2021. Data collected was performed by medical record system and telephone follow-up. The sample used to detect the expression level of hub CRPGs in ELISA assay comes from a prospective study. This study conformed to the Helsinki Declaration and was approved by the Ethics Committee of Guangdong Provincial Hospital of Chinese Medicine. The ethical batch number are ZE2024-027-01 and BF2020-277-02, respectively.

### Participants

The inclusion criteria for patients were as follows: 1. Patients diagnosed with lung cancer by histological or cytological pathological examination; 2. Patients aged 18 years or older; 3. Patients received at least one course of ICIs treatment, whether alone or in combination; 4. Baseline NRS scores were recorded. The exclusion criteria for patients were as follow: 1. Unidentified pathological types, non-primary lesions, or more than two pathological types; 2. Patients with multiple organ primary cancers; 3. Missing follow-up data; 4. Completely missing clinical and laboratory data.

### Intervention

All enrolled lung cancer patients received at least one course of ICIs treatment, either alone or in combination with chemotherapy or targeted therapy. There were no restrictions on the types of ICIs. The determination of whether patients received ICIs treatment was obtained from the medical records system.

### Comparisons

According to NRS scores, the patients were divided into a Pain group (NRS scores > 0) and a Non-Pain group (NRS scores = 0). By comparing the clinical factors between the two groups, we identified key factors affecting the efficacy of ICIs mediated by pain. Kaplan-Meier (KM) survival analysis was used to evaluate the survival differences between the two groups. A P-value of the log-rank test less than 0.05 was considered significant. All tests were two-sided, with an α of 0.05.

### Outcome measures

The main outcome measure is overall survival (OS), defined as the time from the start of ICIs treatment to the occurrence of death.

### Propensity score matching

To mitigate differences between the Pain group and the Non-Pain group, we employed Propensity Score Matching (PSM) analysis. Even with only one confounding variable, we included all variables in the PSM analysis to ensure more reliable results. Nearest neighbor matching was utilized as the matching method, with a caliper value set to 0.05, and a 1:1 ratio of the target group to the control group.

### Cox regression analysis and nomogram construction

Univariate cox analysis was used to identify the prognostic factors affecting the efficacy of ICIs, and multivariate cox analysis was used to identify the independent prognostic risk factors affecting the efficacy of ICIs. The P value of log rank test <0.05 was considered to be significantly different. For the prognostic factors with significant differences in multivariate cox analysis, it was further used to construct nomogram for prognosis evaluation of patients. According to the risk score of the model, KM survival analysis was used to evaluate the difference between them. Finally, ROC curve was used to evaluate the reliability of the model.

### Construction of cancer-related pain matrix

The chip data GSE93157 and platform annotation files GPL19965 of patients with NSCLC receiving ICIs treatment were obtained from the GEO database. This data was published by Prat A et al. in Cancer Research in 2017. The authors utilized the PanCancer 730-Immune Panel to analyze tumor samples from 65 patients treated with anti-PD-1 therapy for melanoma, lung cancer, and head and neck cancer on the nCounter system (29). Among them, there were 13 cases of squamous cell carcinoma and 22 cases of non-small cell non-squamous cell carcinoma, totaling 35 cases. The patients were treated with either NIVOLUMAB or PEMBROLIZUMAB. The targets of cancer-related pain were identified by searching the GeneCards database using the keyword “cancer related pain” and intersecting with genes in the GSE93157 chip. Subsequently, the expression levels of these targets were extracted to create the NSCLC Cancer Related Pain Matrix.

### Consistency cluster analysis and differential expression analysis

Unsupervised learning was conducted utilizing the K-means consensus clustering method to partition the NSCLC Cancer Related Pain Matrix into distinct modules, based on the coherence of internal pain gene expression. Subsequently, the limma package was employed to conduct differential expression analysis between modules, with the threshold set at log |FC (Fold Change)| > 1 and a P value < 0.05.

### GO and KEGG functional enrichment analysis

For the gene set functional enrichment analysis, the KEGG REST API was utilized to access the most recent KEGG Pathway gene annotations and genes in the org.Hs.eg.db. The GO annotation served as the background for the analysis, with DEGs being mapped to this background set. Enrichment analysis was then conducted using the R software package clusterProfiler to determine gene set enrichment outcomes. The minimum gene set size was established at 5, while the maximum gene set size was set at 5000. Significance was determined by a P value of < 0.05 and a false discovery rate (FDR) of < 0.1.

### PPI network construction and correlation analysis

The cancer-related pain genes (CRPGs) from the core KEGG pathway were imported into the STRING database, with Homo sapiens selected as the sample. A correlation coefficient of 0.900 was observed, followed by the utilization of the MCC algorithm within the cytoHubba plug-in of Cytoscape 3.8.2 software to determine the top 10 targets within the core. To further clarify the regulatory relationship between these 10 cytokines, analysis was performed using Pearson correlation analysis.

### Survival analysis

In order to study the survival difference of the top 10 CRPGs in lung cancer patients, the top 10 targets were divided into high and low groups according to their median expression levels. The GEPIA database was used to perform KM survival analysis on LUAD and LUSC respectively. In addition, we used the GEO database lung cancer cohort to externally validate the results in TCGA. Log rank P value <0.05 was considered significant differences.

### Tumor immune dysfunction and exclusion (TIDE) algorithm

In order to predict whether the survival related CRPGs affect the efficacy of immunotherapy for lung cancer, we divided them into two groups, high and low, based on the expression of CRPGs, and used the TIDE algorithm to analyze the effectiveness of ICIs treatment between different groups.

### Correlation analysis between survival related CRPGs, immune cells, and inflammatory pathways

In order to further clarify the immune cells and pathways regulated by survival related CRPGs, the CIBERSORT algorithm and Pearson correlation analysis were used to conduct correlation analysis among the three.

### ELISA assay

The expression levels of hub CRPGs were detected using an enzyme-linked immunosorbent assay (ELISA). ELISA assay was performed using the kit from Lianke Biotechnology (EK1119-AW1). Briefly, 20ul of sample and 80ul of detection buffer were added to the sample well. The subsequent steps were consistent with the instructions. Finally, the OD values at 450nm and 630nm was measured by microplate reader. The CXCL12 content in each sample was calculated from the standard curve.

### Statistical analysis

All data were entered using by WPS Office or Excel, and data analysis and visualization were conducted by R language software 4.2.1 and SPSS version 26.0. Measurement data were assessed for normal distribution and described using the mean standard deviation. For data that did not follow a normal distribution, the median, lower quartile (Q1), upper quartile (Q3), minimum value, and maximum value were reported. The measurement data conform to normal distribution and homogeneity of variance by T test, and those that do not conform to normal distribution or homogeneity of variance by Mann Whitney U test. Counting data were presented as composition ratio and ratio. Chi-square test or Fisher exact probability method was used for analyzing counting data.

For variables with less than 20% missing data, the mice package in R language 4.2.0 was used for data interpolation, and the data interpolation was carried out by setting the number of random seeds. Variables with more than 20% missing data are eliminated.

Univariate Cox analysis was employed to identify prognostic factors associated with ICIs treatment, and multivariate Cox analysis to identify independent prognostic factors associated with ICIs treatment. Independent prognostic factors were utilized to construct a nomogram, and ROC curve analysis was employed to assess the reliability of the model. Additionally, Spearman correlation analysis was used to evaluate the regulatory relationship between NRS and circulating inflammatory cells.

## Results

### Patient characteristics

**Figure 1:**
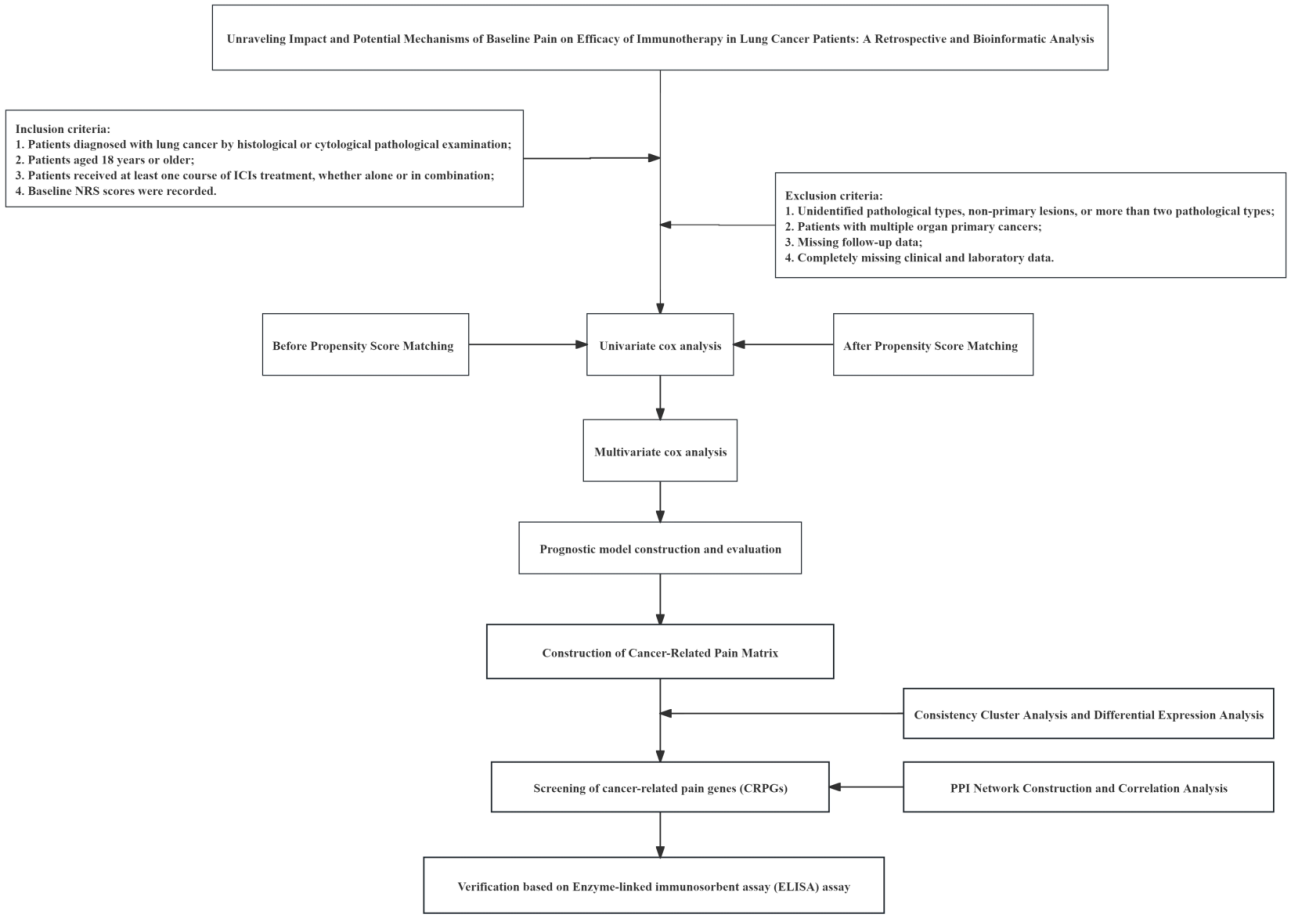
The study workflow.

The study workflow was shown in **Figure 1**. Before PSM, a total of 222 patients with lung cancer treated with ICIs were enrolled. Patients were divided into Pain group and Non-Pain group according to NRS scores. There were 112 cases in Pain group and 110 cases in Non-Pain group. The difference analysis between groups showed that PLR, HBV, M stage, Bone metastasis and pleural metastasis in Pain group were significantly higher than those in Non-Pain group. The detail of baseline before PSM was shown in **Table 1**.

**Table 1:** The detail of baseline before PSM.

Variable	Total (n = 222)	Group		Statistic		
		Non-Pain (n = 110)	Pain (n = 112)	Z	P	SMD
Age, M (Q_1_, Q_3_)	62.00 (56.00, 67.00)	63.00 (56.25, 67.75)	62.00 (56.00, 67.00)	Z=-1.337	0.181	-0.205
Weight, M (Q_1_, Q_3_)	58.00 (52.00, 65.00)	58.00 (52.00, 64.00)	58.00 (51.75, 65.75)	Z=-0.100	0.920	0.047
Height, M (Q_1_, Q_3_)	165.00 (160.00, 170.00)	165.00 (160.00, 170.00)	165.00 (160.00, 170.00)	Z=-0.569	0.570	0.077
BSA, M (Q_1_, Q_3_)	1.63 (1.55, 1.73)	1.63 (1.56, 1.74)	1.62 (1.55, 1.72)	Z=-0.074	0.941	0.040
Neutrophils, M (Q_1_, Q_3_)	5.10 (3.82, 6.86)	5.11 (3.82, 6.44)	5.08 (3.80, 6.89)	Z=-0.077	0.938	0.059
Lymphocytes, M (Q_1_, Q_3_)	1.37 (1.02, 1.81)	1.43 (1.10, 1.81)	1.31 (0.93, 1.80)	Z=-1.401	0.161	-0.112
Leukocytes, M (Q_1_, Q_3_)	7.45 (5.89, 9.70)	7.54 (6.13, 9.46)	7.39 (5.74, 10.02)	Z=-0.524	0.601	0.029
Platelets, M (Q_1_, Q_3_)	279.50 (216.50, 348.25)	276.50 (214.00, 324.75)	286.50 (223.00, 358.25)	Z=-1.101	0.271	0.176
Monocytes, M (Q_1_, Q_3_)	0.56 (0.39, 0.81)	0.54 (0.41, 0.76)	0.57 (0.38, 0.86)	Z=-0.474	0.635	0.112
NLR, M (Q_1_, Q_3_)	3.80 (2.62, 6.12)	3.62 (2.53, 5.46)	4.01 (2.64, 6.26)	Z=-1.003	0.316	-1.267
Dnlr, M (Q_1_, Q_3_)	2.33 (1.75, 3.16)	2.25 (1.76, 2.92)	2.44 (1.75, 3.35)	Z=-1.329	0.184	0.167
PLR, M (Q_1_, Q_3_)	203.56 (150.05, 295.17)	185.00 (148.36, 231.87)	213.02 (156.47, 321.33)	Z=-2.419	**0.016**	0.295
LMR, M (Q_1_, Q_3_)	2.36 (1.63, 3.35)	2.46 (1.64, 3.45)	2.27 (1.61, 3.16)	Z=-0.807	0.420	-0.011
SII, M (Q_1_, Q_3_)	1036.97 (625.13, 1621.41)	922.05 (600.19, 1555.31)	1113.73 (728.34, 1695.25)	Z=-1.717	0.086	0.166
Gender, n (%)				χ²=0.197	0.657	
1	179 (80.63)	90 (81.82)	89 (79.46)			-0.058
2	43 (19.37)	20 (18.18)	23 (20.54)			0.058
Alcohol, n (%)				χ²=1.763	0.184	
0	168 (75.68)	79 (71.82)	89 (79.46)			0.189
1	54 (24.32)	31 (28.18)	23 (20.54)			-0.189
ECOG, n (%)				-	0.137	
0	100 (45.05)	57 (51.82)	43 (38.39)			-0.276
1	100 (45.05)	45 (40.91)	55 (49.11)			0.164
2	12 (5.41)	6 (5.45)	6 (5.36)			-0.004
3	8 (3.6)	2 (1.82)	6 (5.36)			0.157
4	2 (0.9)	0 (0.00)	2 (1.79)			0.135
Smoke, n (%)				χ²=0.681	0.409	
0	60 (27.03)	27 (24.55)	33 (29.46)			0.108
1	162 (72.97)	83 (75.45)	79 (70.54)			-0.108
HBV, n (%)				χ²=4.487	**0.034**	
0	203 (91.44)	105 (95.45)	98 (87.50)			-0.241
1	19 (8.56)	5 (4.55)	14 (12.50)			0.241
Tumor, n (%)				χ²=0.889	0.346	
1	200 (90.09)	97 (88.18)	103 (91.96)			0.139
2	22 (9.91)	13 (11.82)	9 (8.04)			-0.139
Pathology, n (%)				χ²=1.971	0.373	
1	125 (56.31)	57 (51.82)	68 (60.71)			0.182
2	55 (24.77)	29 (26.36)	26 (23.21)			-0.075
3	42 (18.92)	24 (21.82)	18 (16.07)			-0.156
T, n (%)				χ²=4.341	0.362	
0	30 (13.51)	17 (15.45)	13 (11.61)			-0.120
1	25 (11.26)	10 (9.09)	15 (13.39)			0.126
2	36 (16.22)	16 (14.55)	20 (17.86)			0.086
3	30 (13.51)	19 (17.27)	11 (9.82)			-0.250
4	101 (45.5)	48 (43.64)	53 (47.32)			0.074
N, n (%)				-	0.958	
0	40 (18.02)	19 (17.27)	21 (18.75)			0.038
1	3 (1.35)	2 (1.82)	1 (0.89)			-0.098
2	85 (38.29)	43 (39.09)	42 (37.50)			-0.033
3	94 (42.34)	46 (41.82)	48 (42.86)			0.021
M, n (%)				χ²=14.810	**<.001**	
0	47 (21.17)	35 (31.82)	12 (10.71)			-0.682
1	175 (78.83)	75 (68.18)	100 (89.29)			0.682
Lung metastasis, n (%)				χ²=1.020	0.313	
0	140 (63.06)	73 (66.36)	67 (59.82)			-0.133
1	82 (36.94)	37 (33.64)	45 (40.18)			0.133
Liver metastasis, n (%)				χ²=1.801	0.180	
0	193 (86.94)	99 (90.00)	94 (83.93)			-0.165
1	29 (13.06)	11 (10.00)	18 (16.07)			0.165
Bone metastasis, n (%)				χ²=36.352	**<.001**	
0	131 (59.01)	87 (79.09)	44 (39.29)			-0.815
1	91 (40.99)	23 (20.91)	68 (60.71)			0.815
Brain metastasis, n (%)				χ²=3.681	0.055	
0	176 (79.28)	93 (84.55)	83 (74.11)			-0.238
1	46 (20.72)	17 (15.45)	29 (25.89)			0.238
Adrenal metastasis, n (%)				χ²=0.004	0.950	
0	182 (81.98)	90 (81.82)	92 (82.14)			0.008
1	40 (18.02)	20 (18.18)	20 (17.86)			-0.008
Other Lymph node metastasis, n (%)				χ²=0.744	0.388	
0	187 (84.23)	95 (86.36)	92 (82.14)			-0.110
1	35 (15.77)	15 (13.64)	20 (17.86)			0.110
Pleura metastasis, n (%)				χ²=6.242	**0.012**	
0	172 (77.48)	93 (84.55)	79 (70.54)			-0.307
1	50 (22.52)	17 (15.45)	33 (29.46)			0.307
Meningeal metastasis, n (%)				χ²=0.000	1.000	
0	213 (95.95)	106 (96.36)	107 (95.54)			-0.040
1	9 (4.05)	4 (3.64)	5 (4.46)			0.040

Bold values: P value<0.05.

After PSM, a total of 94 patients with lung cancer treated with ICIs were enrolled. There were 47 cases in Pain Group and 47 cases in Non-Pain group. PSM eliminated the confounding factors between groups, and there was no significant difference in all included clinical indexes. The detail of baseline after PSM was shown in **Table 2**.

**Table 2:** The detail of baseline after PSM.

Variable	Total (n = 94)	Group		Statistic		
		Non-Pain (n = 47)	Pain (n = 47)	Z	P	SMD
Age, M (Q_1_, Q_3_)	62.00 (54.00, 66.00)	61.00 (53.00, 67.00)	62.00 (56.00, 66.00)	Z=-0.413	0.680	0.002
Weight, M (Q_1_, Q_3_)	59.00 (54.00, 65.00)	58.50 (52.00, 65.75)	59.50 (56.00, 64.50)	Z=-0.908	0.364	0.181
Height, M (Q_1_, Q_3_)	168.00 (160.00, 170.00)	165.00 (159.00, 170.00)	168.00 (160.50, 170.50)	Z=-0.921	0.357	0.174
BSA, M (Q_1_, Q_3_)	1.65 (1.57, 1.74)	1.63 (1.56, 1.76)	1.66 (1.57, 1.73)	Z=-0.969	0.333	0.282
Neutrophils, M (Q_1_, Q_3_)	4.66 (3.30, 6.46)	5.01 (3.72, 6.75)	4.49 (3.05, 6.37)	Z=-0.866	0.387	-0.084
Lymphocytes, M (Q_1_, Q_3_)	1.44 (1.10, 1.84)	1.40 (1.11, 1.75)	1.53 (1.09, 1.93)	Z=-0.609	0.543	0.209
Leukocytes, M (Q_1_, Q_3_)	7.21 (5.38, 9.32)	7.25 (5.62, 9.64)	6.77 (5.04, 8.73)	Z=-0.650	0.515	-0.001
Platelets, M (Q_1_, Q_3_)	272.50 (213.25, 321.75)	266.00 (213.50, 322.00)	281.00 (217.00, 321.50)	Z=-0.386	0.700	0.089
Monocytes, M (Q_1_, Q_3_)	0.54 (0.37, 0.72)	0.54 (0.36, 0.68)	0.55 (0.38, 0.83)	Z=-0.495	0.620	0.114
NLR, M (Q_1_, Q_3_)	3.56 (2.28, 5.43)	3.89 (2.35, 5.46)	3.38 (2.25, 5.06)	Z=-0.873	0.382	-0.304
Dnlr, M (Q_1_, Q_3_)	2.12 (1.56, 3.04)	2.26 (1.65, 3.06)	1.98 (1.60, 2.82)	Z=-0.945	0.345	-0.335
PLR, M (Q_1_, Q_3_)	189.09 (146.99, 233.83)	194.44 (151.81, 259.75)	188.89 (138.38, 227.18)	Z=-1.157	0.247	-0.176
LMR, M (Q_1_, Q_3_)	2.54 (1.74, 3.53)	2.50 (1.71, 3.38)	2.54 (1.80, 3.69)	Z=-0.635	0.525	0.071
SII, M (Q_1_, Q_3_)	870.69 (581.38, 1465.57)	988.13 (592.40, 1605.02)	820.99 (571.88, 1411.20)	Z=-0.873	0.382	-0.074
Gender, n (%)				χ²=0.275	0.600	
1	76 (80.85)	39 (82.98)	37 (78.72)			-0.104
2	18 (19.15)	8 (17.02)	10 (21.28)			0.104
Alcohol, n (%)				χ²=2.014	0.156	
0	70 (74.47)	32 (68.09)	38 (80.85)			0.324
1	24 (25.53)	15 (31.91)	9 (19.15)			-0.324
ECOG, n (%)				-	0.180	
0	44 (46.81)	23 (48.94)	21 (44.68)			-0.086
1	43 (45.74)	18 (38.30)	25 (53.19)			0.298
2	5 (5.32)	4 (8.51)	1 (2.13)			-0.442
3	2 (2.13)	2 (4.26)	0 (0.00)			-0.298
Smoke, n (%)				χ²=0.203	0.652	
0	28 (29.79)	13 (27.66)	15 (31.91)			0.091
1	66 (70.21)	34 (72.34)	32 (68.09)			-0.091
HBV, n (%)				χ²=0.261	0.609	
0	90 (95.74)	44 (93.62)	46 (97.87)			0.295
1	4 (4.26)	3 (6.38)	1 (2.13)			-0.295
Tumor, n (%)				χ²=0.103	0.748	
1	83 (88.3)	41 (87.23)	42 (89.36)			0.069
2	11 (11.7)	6 (12.77)	5 (10.64)			-0.069
Pathology, n (%)				χ²=1.554	0.460	
1	46 (48.94)	20 (42.55)	26 (55.32)			0.257
2	28 (29.79)	16 (34.04)	12 (25.53)			-0.195
3	20 (21.28)	11 (23.40)	9 (19.15)			-0.108
T, n (%)				χ²=1.022	0.907	
0	18 (19.15)	9 (19.15)	9 (19.15)			0.000
1	9 (9.57)	5 (10.64)	4 (8.51)			-0.076
2	15 (15.96)	6 (12.77)	9 (19.15)			0.162
3	11 (11.7)	5 (10.64)	6 (12.77)			0.064
4	41 (43.62)	22 (46.81)	19 (40.43)			-0.130
N, n (%)				χ²=0.377	0.828	
0	14 (14.89)	6 (12.77)	8 (17.02)			0.113
2	36 (38.3)	18 (38.30)	18 (38.30)			0.000
3	44 (46.81)	23 (48.94)	21 (44.68)			-0.086
M, n (%)				χ²=0.072	0.789	
0	17 (18.09)	8 (17.02)	9 (19.15)			0.054
1	77 (81.91)	39 (82.98)	38 (80.85)			-0.054
Lung metastasis, n (%)				χ²=0.720	0.396	
0	58 (61.7)	27 (57.45)	31 (65.96)			0.180
1	36 (38.3)	20 (42.55)	16 (34.04)			-0.180
Liver metastasis, n (%)				χ²=2.574	0.109	
0	83 (88.3)	39 (82.98)	44 (93.62)			0.435
1	11 (11.7)	8 (17.02)	3 (6.38)			-0.435
Bone metastasis, n (%)				χ²=0.048	0.826	
0	63 (67.02)	32 (68.09)	31 (65.96)			-0.045
1	31 (32.98)	15 (31.91)	16 (34.04)			0.045
Brain metastasis, n (%)				χ²=0.066	0.797	
0	75 (79.79)	38 (80.85)	37 (78.72)			-0.052
1	19 (20.21)	9 (19.15)	10 (21.28)			0.052
Adrenal metastasis, n (%)				χ²=0.072	0.789	
0	77 (81.91)	39 (82.98)	38 (80.85)			-0.054
1	17 (18.09)	8 (17.02)	9 (19.15)			0.054
Other Lymph node metastasis, n (%)				χ²=0.000	1.000	
0	76 (80.85)	38 (80.85)	38 (80.85)			0.000
1	18 (19.15)	9 (19.15)	9 (19.15)			0.000
Pleura metastasis, n (%)				χ²=0.066	0.797	
0	75 (79.79)	37 (78.72)	38 (80.85)			0.054
1	19 (20.21)	10 (21.28)	9 (19.15)			-0.054
Meningeal metastasis, n (%)				χ²=0.000	1.000	
0	92 (97.87)	46 (97.87)	46 (97.87)			0.000
1	2 (2.13)	1 (2.13)	1 (2.13)			0.000

Bold values: P value<0.05.

### Univariate cox analysis

Before PSM, univariate cox analysis suggested that Dnlr (HR: 1.10, 95% CI: 1.01 - 1.21, p value: 0.027), PLR (HR: 1.01, 95% CI: 1.01 - 1.01, p value: 0.002), Systemic inflammation index (SII) (HR: 1.01, 95% CI: 1.01 - 1.01, p value: 0.014), Eastern Cooperative Oncology Group (ECOG) scores (HR: 13.55, 95% CI: 3.18 - 57.68, p value < 0.001, 4 vs 0), M stage (HR: 1.52, 95% CI: 1.03 - 2.24, p value: 0.033), Lung metastasis (HR: 1.47, 95% CI: 1.08 - 2.00, p value: 0.015), Bone metastasis (HR: 1.76, 95% CI: 1.30 - 2.38, p value < 0.001), Adrenal metastasis (HR: 1.51, 95% CI: 1.04 - 2.19, p value: 0.031) and NRS score (HR: 1.65, 95% CI: 1.21 - 2.23, p value: 0.001) were risk factors of ICIs. **Table 3**.

**Table 3:** Univariate cox analysis and Multivariate cox analysis before PSM.

Variables	Beta	S.E	Z	P	HR (95%CI)	m_Beta	m_S.E	m_Z	aP	aHR (95%CI)
Age	0.01	0.01	0.87	0.387	1.01 (0.99 - 1.03)	0.01	0.01	0.67	0.500	1.01 (0.99 - 1.03)
Weight	-0.01	0.01	-1.49	0.137	0.99 (0.97 - 1.00)	-0.04	0.05	-0.93	0.353	0.96 (0.88 - 1.05)
Height	0.01	0.01	0.77	0.443	1.01 (0.99 - 1.03)	0.00	0.03	0.03	0.973	1.00 (0.94 - 1.07)
BSA	-0.55	0.57	-0.97	0.334	0.58 (0.19 - 1.76)	2.43	3.84	0.63	0.527	11.36 (0.01 - 21151.82)
Neutrophils	0.03	0.03	1.00	0.317	1.03 (0.98 - 1.08)	-0.26	0.33	-0.79	0.431	0.77 (0.40 - 1.47)
Lymphocytes	-0.23	0.12	-1.91	0.057	0.80 (0.63 - 1.01)	-0.09	0.35	-0.26	0.794	0.91 (0.46 - 1.82)
Leukocyte	0.01	0.02	0.54	0.592	1.01 (0.97 - 1.06)	0.16	0.30	0.53	0.594	1.17 (0.65 - 2.10)
Platelets	0.00	0.00	1.20	0.229	1.00 (1.00 - 1.00)	0.00	0.00	0.14	0.887	1.00 (1.00 - 1.00)
Monocytes	0.30	0.24	1.26	0.208	1.35 (0.85 - 2.16)	0.73	0.52	1.41	0.160	2.08 (0.75 - 5.79)
NLR	0.00	0.00	1.74	0.082	1.00 (1.00 - 1.01)	0.00	0.00	1.02	0.310	1.00 (1.00 - 1.01)
Dnlr	0.10	0.05	2.20	**0.027**	1.10 (1.01 - 1.21)	0.19	0.15	1.24	0.215	1.21 (0.90 - 1.62)
PLR	0.01	0.00	3.02	**0.002**	1.01 (1.01 - 1.01)	0.00	0.00	0.56	0.574	1.00 (1.00 - 1.00)
LMR	-0.06	0.04	-1.51	0.131	0.94 (0.88 - 1.02)	0.02	0.05	0.43	0.670	1.02 (0.92 - 1.13)
SII	0.01	0.00	2.45	**0.014**	1.01 (1.01 - 1.01)	0.00	0.00	0.09	0.928	1.00 (1.00 - 1.00)
Gender										
1					Ref					Ref
2	0.11	0.19	0.57	0.566	1.11 (0.77 - 1.61)	0.54	0.37	1.46	0.145	1.72 (0.83 - 3.55)
Alcohol										
0					Ref					Ref
1	-0.32	0.19	-1.72	0.086	0.73 (0.50 - 1.05)	-0.13	0.24	-0.53	0.593	0.88 (0.55 - 1.41)
ECOG										
0					Ref					Ref
1	0.10	0.16	0.59	0.556	1.10 (0.80 - 1.51)	-0.00	0.20	-0.01	0.995	1.00 (0.68 - 1.47)
2	0.32	0.35	0.91	0.361	1.38 (0.69 - 2.76)	0.03	0.43	0.07	0.941	1.03 (0.45 - 2.38)
3	0.76	0.40	1.91	0.056	2.14 (0.98 - 4.65)	0.29	0.61	0.48	0.634	1.34 (0.40 - 4.42)
4	2.61	0.74	3.53	**<.001**	13.55 (3.18 - 57.68)	1.34	1.02	1.31	0.189	3.83 (0.52 - 28.41)
Smoke										
0					Ref					Ref
1	-0.13	0.17	-0.76	0.450	0.88 (0.63 - 1.23)	-0.04	0.30	-0.12	0.904	0.96 (0.53 - 1.75)
HBV										
0					Ref					Ref
1	0.06	0.26	0.23	0.815	1.06 (0.64 - 1.76)	-0.21	0.32	-0.64	0.521	0.81 (0.43 - 1.53)
Tumor										
1					Ref					Ref
2	0.36	0.26	1.41	0.158	1.44 (0.87 - 2.38)	0.40	0.41	0.97	0.331	1.49 (0.66 - 3.36)
Lung pathology										
1					Ref					Ref
2	0.08	0.19	0.44	0.663	1.09 (0.75 - 1.57)	0.45	0.24	1.89	0.059	1.57 (0.98 - 2.50)
3	0.25	0.20	1.26	0.208	1.29 (0.87 - 1.90)	0.29	0.29	0.98	0.328	1.33 (0.75 - 2.37)
T										
0					Ref					Ref
1	-0.48	0.32	-1.47	0.141	0.62 (0.33 - 1.17)	-0.50	0.38	-1.31	0.189	0.60 (0.28 - 1.28)
2	-0.01	0.28	-0.03	0.975	0.99 (0.57 - 1.71)	-0.16	0.37	-0.42	0.672	0.86 (0.42 - 1.76)
3	-0.02	0.30	-0.06	0.949	0.98 (0.55 - 1.75)	0.09	0.39	0.23	0.816	1.10 (0.51 - 2.35)
4	0.06	0.23	0.25	0.802	1.06 (0.67 - 1.68)	0.01	0.30	0.03	0.975	1.01 (0.56 - 1.82)
N										
0					Ref					Ref
1	-0.23	0.73	-0.32	0.749	0.79 (0.19 - 3.32)	-0.22	0.82	-0.27	0.788	0.80 (0.16 - 3.98)
2	0.02	0.22	0.08	0.935	1.02 (0.66 - 1.58)	-0.07	0.27	-0.25	0.802	0.93 (0.55 - 1.60)
3	0.13	0.22	0.59	0.556	1.14 (0.74 - 1.75)	-0.02	0.28	-0.05	0.956	0.98 (0.57 - 1.71)
M										
0					Ref					Ref
1	0.42	0.20	2.13	**0.033**	1.52 (1.03 - 2.24)	-0.07	0.28	-0.23	0.816	0.94 (0.54 - 1.63)
Lung metastasis										
0					Ref					Ref
1	0.38	0.16	2.43	**0.015**	1.47 (1.08 - 2.00)	0.35	0.21	1.67	0.095	1.41 (0.94 - 2.12)
Liver metastasis										
0					Ref					Ref
1	0.27	0.22	1.25	0.213	1.31 (0.86 - 2.01)	0.25	0.30	0.84	0.398	1.28 (0.72 - 2.29)
Bone metastasis										
0					Ref					Ref
1	0.56	0.16	3.62	**<.001**	1.76 (1.30 - 2.38)	0.49	0.22	2.20	**0.028**	1.63 (1.05 - 2.51)
Brain metastasis										
0					Ref					Ref
1	0.17	0.18	0.94	0.348	1.19 (0.83 - 1.71)	-0.11	0.24	-0.46	0.648	0.90 (0.56 - 1.44)
Adrenal metastasis										
0					Ref					Ref
1	0.41	0.19	2.15	**0.031**	1.51 (1.04 - 2.19)	0.27	0.25	1.07	0.283	1.31 (0.80 - 2.16)
Other Lymph node metastasis										
0					Ref					Ref
1	0.17	0.21	0.82	0.414	1.19 (0.79 - 1.78)	-0.05	0.28	-0.18	0.855	0.95 (0.54 - 1.65)
Pleura metastasis										
0					Ref					Ref
1	0.36	0.18	1.96	0.050	1.43 (1.01 - 2.04)	0.16	0.25	0.64	0.522	1.17 (0.72 - 1.91)
Meningeal metastasis										
0					Ref					Ref
1	-0.21	0.42	-0.50	0.614	0.81 (0.36 - 1.83)	-0.23	0.55	-0.43	0.668	0.79 (0.27 - 2.31)
NRS										
0					Ref					Ref
1	0.50	0.16	3.20	**0.001**	1.65 (1.21 - 2.23)	0.45	0.20	2.27	**0.023**	1.56 (1.06 - 2.30)

Bold values: P value<0.05.

After PSM, univariate cox analysis suggested that Age (HR: 1.04, 95% CI: 1.01 - 1.07, p value: 0.012), NLR (HR: 1.09, 95% CI: 1.03 - 1.17, p value: 0.006), Derived neutrophil lymphocyte ratio (Dnlr) (HR: 1.18, 95% CI: 1.03 - 1.36, p value: 0.017), PLR (HR: 1.01, 95% CI: 1.01 - 1.01, p value: 0.030), SII (HR: 1.01, 95% CI: 1.01 - 1.01, p value: 0.009), Bone metastasis (HR: 1.98, 95% CI: 1.21 - 3.24, p value: 0.006) were risk factors of ICIs. **Table 4**.

**Table 4:** Univariate cox analysis and Multivariate cox analysis after PSM.

Variables	Univariate					Multivariate				
	β	S. E	t	P	HR (95%CI)	β	S. E	t	P	HR (95%CI)
Age	0.04	0.01	2.52	**0.012**	1.04 (1.01 - 1.07)	0.08	0.02	3.35	**<.001**	1.08 (1.03 - 1.13)
Weight	-0.02	0.01	-1.28	0.201	0.98 (0.96 - 1.01)	-0.04	0.12	-0.31	0.759	0.96 (0.77 - 1.21)
Height	0.02	0.02	0.97	0.331	1.02 (0.98 - 1.05)	0.01	0.07	0.10	0.917	1.01 (0.88 - 1.15)
BSA	-0.38	0.87	-0.44	0.661	0.68 (0.13 - 3.73)	4.50	9.10	0.49	0.621	89.62 (0.00 - 4947835799.89)
Neutrophils	0.03	0.03	0.84	0.400	1.03 (0.96 - 1.10)	-1.22	0.72	-1.70	0.089	0.30 (0.07 - 1.20)
Lymphocytes	-0.27	0.18	-1.49	0.135	0.77 (0.54 - 1.09)	-1.94	1.00	-1.93	0.054	0.14 (0.02 - 1.03)
Leukocyte	0.02	0.03	0.57	0.568	1.02 (0.96 - 1.08)	1.09	0.66	1.64	0.100	2.96 (0.81 - 10.84)
Platelets	0.00	0.00	0.95	0.343	1.00 (1.00 - 1.00)	0.01	0.00	2.74	**0.006**	1.01 (1.01 - 1.02)
Monocytes	0.31	0.33	0.94	0.348	1.36 (0.72 - 2.58)	0.79	1.01	0.78	0.438	2.20 (0.30 - 16.05)
NLR	0.09	0.03	2.77	**0.006**	1.09 (1.03 - 1.17)	0.01	0.13	0.09	0.926	1.01 (0.79 - 1.30)
Dnlr	0.17	0.07	2.38	**0.017**	1.18 (1.03 - 1.36)	1.18	0.46	2.57	**0.010**	3.27 (1.33 - 8.04)
PLR	0.01	0.00	2.16	**0.030**	1.01 (1.01 - 1.01)	-0.00	0.01	-0.70	0.484	1.00 (0.99 - 1.01)
LMR	-0.11	0.06	-1.71	0.087	0.90 (0.80 - 1.02)	0.11	0.11	1.01	0.312	1.12 (0.90 - 1.39)
SII	0.01	0.00	2.62	**0.009**	1.01 (1.01 - 1.01)	-0.00	0.00	-1.95	0.051	1.00 (1.00 - 1.00)
Gender										
1					Ref					Ref
2	0.05	0.29	0.16	0.873	1.05 (0.59 - 1.85)	0.53	0.76	0.70	0.486	1.69 (0.38 - 7.47)
Alcohol										
0					Ref					Ref
1	-0.19	0.27	-0.68	0.495	0.83 (0.49 - 1.42)	0.35	0.53	0.67	0.501	1.43 (0.51 - 4.00)
ECOG										
0					Ref					Ref
1	0.16	0.25	0.63	0.527	1.17 (0.72 - 1.90)	0.49	0.40	1.24	0.215	1.63 (0.75 - 3.54)
2	0.51	0.48	1.06	0.291	1.66 (0.65 - 4.27)	0.58	1.08	0.54	0.592	1.79 (0.21 - 14.89)
3		0.00	NA		NA (NA - NA)		0.00	NA		NA (NA - NA)
Smoke										
0					Ref					Ref
1	-0.00	0.26	-0.00	0.999	1.00 (0.61 - 1.65)	-0.15	0.64	-0.24	0.809	0.86 (0.25 - 3.00)
HBV										
0					Ref					Ref
1	0.10	0.52	0.19	0.849	1.10 (0.40 - 3.03)	0.66	0.73	0.90	0.366	1.93 (0.46 - 8.00)
Tumor										
1					Ref					Ref
2	0.33	0.36	0.93	0.354	1.39 (0.69 - 2.80)	-0.16	0.78	-0.20	0.842	0.86 (0.19 - 3.94)
Pathology										
1					Ref					Ref
2	-0.13	0.28	-0.46	0.645	0.88 (0.51 - 1.52)	0.50	0.46	1.07	0.283	1.64 (0.66 - 4.06)
3	0.28	0.30	0.93	0.350	1.32 (0.74 - 2.35)	0.20	0.58	0.35	0.729	1.22 (0.39 - 3.80)
T										
0					Ref					Ref
1	-0.96	0.52	-1.84	0.066	0.38 (0.14 - 1.07)	-0.31	0.80	-0.38	0.701	0.74 (0.15 - 3.52)
2	-0.26	0.40	-0.64	0.519	0.77 (0.35 - 1.69)	-0.75	0.62	-1.21	0.228	0.47 (0.14 - 1.60)
3	0.05	0.42	0.12	0.907	1.05 (0.46 - 2.40)	0.30	0.63	0.48	0.632	1.35 (0.40 - 4.61)
4	0.00	0.31	0.01	0.990	1.00 (0.54 - 1.85)	-0.01	0.49	-0.02	0.985	0.99 (0.38 - 2.59)
N										
0					Ref					Ref
2	-0.09	0.36	-0.25	0.803	0.91 (0.45 - 1.84)	-0.06	0.56	-0.10	0.921	0.95 (0.32 - 2.82)
3	-0.16	0.35	-0.46	0.645	0.85 (0.43 - 1.69)	-0.87	0.61	-1.43	0.152	0.42 (0.13 - 1.38)
M										
0					Ref					Ref
1	0.07	0.31	0.24	0.807	1.08 (0.59 - 1.96)	-0.32	0.53	-0.59	0.552	0.73 (0.26 - 2.07)
Lung metastasis										
0					Ref					Ref
1	0.30	0.24	1.24	0.215	1.34 (0.84 - 2.15)	0.84	0.47	1.76	0.078	2.31 (0.91 - 5.85)
Liver metastasis										
0					Ref					Ref
1	0.19	0.34	0.56	0.577	1.21 (0.62 - 2.36)	1.35	0.59	2.29	**0.022**	3.88 (1.22 - 12.34)
Bone metastasis										
0					Ref					Ref
1	0.68	0.25	2.73	**0.006**	1.98 (1.21 - 3.24)	1.30	0.47	2.78	**0.005**	3.66 (1.47 - 9.16)
Brain metastasis										
0					Ref					Ref
1	0.22	0.29	0.75	0.452	1.24 (0.71 - 2.18)	0.01	0.51	0.03	0.980	1.01 (0.37 - 2.76)
Adrenal metastasis										
0					Ref					Ref
1	0.40	0.29	1.37	0.172	1.49 (0.84 - 2.65)	-0.29	0.50	-0.57	0.567	0.75 (0.28 - 2.01)
Other Lymph node metastasis										
0					Ref					Ref
1	-0.24	0.30	-0.80	0.426	0.78 (0.43 - 1.42)	-0.00	0.50	-0.01	0.995	1.00 (0.37 - 2.66)
Pleura metastasis										
0					Ref					Ref
1	0.40	0.29	1.38	0.167	1.50 (0.84 - 2.65)	0.34	0.45	0.76	0.448	1.40 (0.58 - 3.37)
Meningeal metastasis										
0					Ref					Ref
1	-17.07	3113.62	-0.01	0.996	0.00 (0.00 - Inf)	-17.61	3344.31	-0.01	0.996	0.00 (0.00 - Inf)
NRS										
0					Ref					Ref
1	0.40	0.24	1.68	0.093	1.49 (0.94 - 2.36)	0.98	0.36	2.74	**0.006**	2.66 (1.32 - 5.37)

Bold values: P value<0.05.

### Multivariate cox analysis

Before PSM, Multivariate cox analysis suggested that Bone metastasis (HR: 1.63, 95% CI: 1.05 - 2.51, p value: 0.028) and NRS scores (HR: 1.56, 95% CI: 1.06 - 2.30, p value: 0.023) were independent prognostic factors for the efficacy of ICIs. **Table 3**.

After PSM, Multivariate cox analysis suggested that Age (HR: 1.08, 95% CI: 1.03 - 1.13, p value< 0.001), Platelets (HR: 1.01, 95% CI: 1.01 - 1.02, p value: 0.006), Dnlr (HR: 3.27, 95% CI: 1.33 - 8.04, p value: 0.010), Liver metastasis (HR: 3.88, 95% CI: 1.22 - 12.34, p value: 0.022), Bone metastasis (HR: 3.66, 95% CI: 1.47 - 9.16, p value: 0.005) and NRS score (HR: 2.66, 95% CI: 1.32 - 5.37, p value: 0.006) were independent prognostic factors for the efficacy of ICIs. **Table 4**.

### Prognostic model construction and evaluation

**Figure 2:**
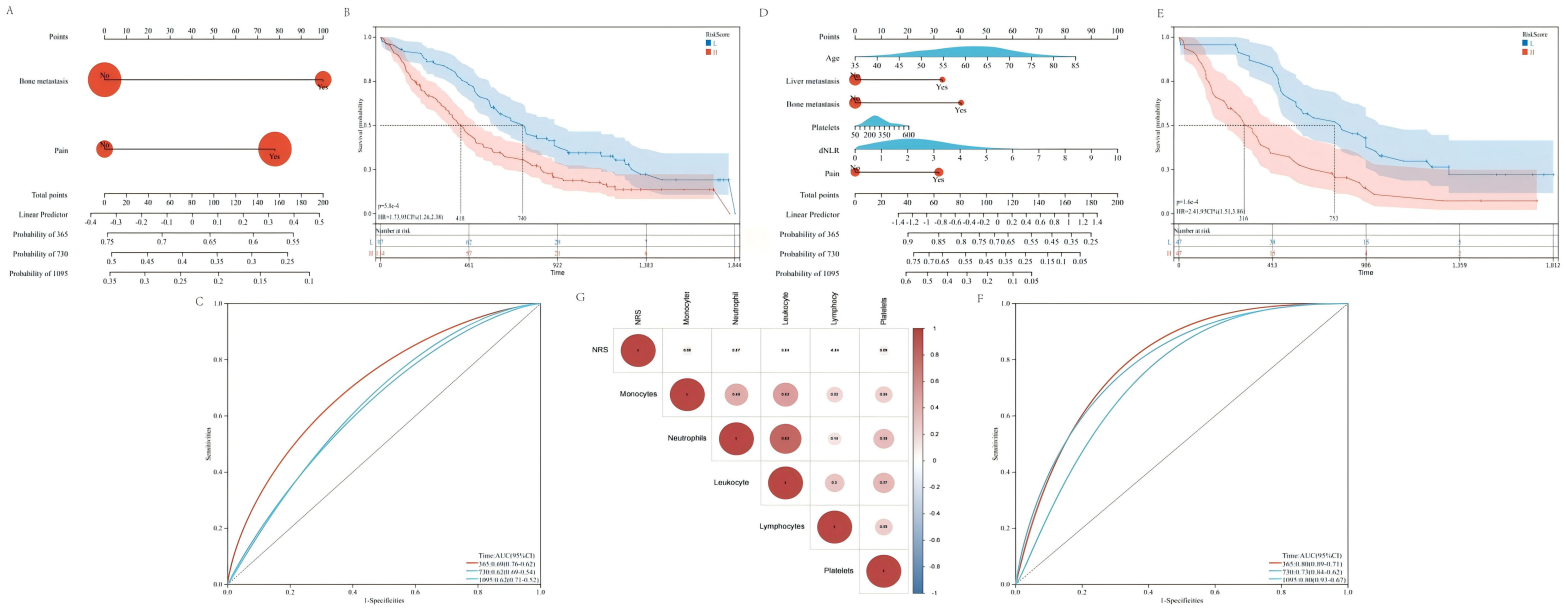
Cox regression analysis and correlation analysis indicated that baseline pain was the independent prognostic factor for ICIs treatment. **(A)** Nomogram construction based on bone metastasis and pain before PSM. **(B)** KM survival analysis showed that the prognosis of low risk patients better than high risk patients (HR=1.73, 95%CI: 1.26-2.38, p=5.8e-4). **(C)** The AUC under the ROC curve with 1 year 0.69, 3 year 0.62 and 5year 0.62. **(D)** Nomogram construction based on age, liver metastasis, bone metastasis, platelets, dNLR and pain after PSM. **(E)** KM survival analysis showed that the prognosis of low risk patients better than high risk patients (HR=2.41, 95%CI: 1.51-3.86, p=1.6e-4). **(F)** The AUC under the ROC curve with 1 year 0.80, 3 year 0.73 and 5year 0.80. **(G)** Correlation analysis showed that NRS score was positively correlated with monocytes, neutrophils, leukocytes and platelets, and negatively correlated with lymphocytes.

Before PSM, based on bone metastasis and NRS scores, we established nomogram for prognosis evaluation of patients **Figure 2A**. It can be seen that patients with lung cancer complicated with Bone metastasis and pain have poor effect and prognosis after receiving ICIs treatment. The survival of lung cancer patients with low risk score is significantly better than that with High risk score. The median OS was 740 days and 418 days respectively, HR=1.73,95CI% (1.26, 2.38), and P value: 5.8e-4. **Figure 2B**. Nomogram’s predictive efficiency was good with AUC under roc curve 1-year 0.69, 2-year 0.62 and 3-year 0.62 **Figure 2C**.

After PSM, based on age, platelets, dNLR, liver metastasis, bone metastasis and NRS Scores, we established nomogram for prognosis evaluation of patients **Figure 2D**. It can be seen that patients with liver metastasis and bone metastasis, high platelet and dNLR, and pain have a poor prognosis after receiving ICIs treatment. The survival of lung cancer patients with Low risk score is significantly better than that with High risk score. The median OS was 753 days and 316 days respectively, HR=2.41,95CI%(1.51,3.86), and P value: 1.6e-4 **Figure 2E**. Nomogram’s predictive efficiency was good with AUC under roc curve 1-year 0.80, 2-year 0.73 and 3-year 0.80 **Figure 2F**. Whether before or after PSM, the risk score showed significant different between with or without bone metastasis and pain group **Supplementary Figures 1A–D**. And with the increasing of age and dNLR, the risk scores was also increased **Supplementary Figures 1E, F**.

### Spearman correlation analysis among NRS scores and peripheral circulating inflammatory cells

Spearman correlation analysis suggested that NRS had positive regulation on monocytes (cor: 0.08), neutrophils (cor: 0.07), leukocytes (cor: 0.04) and platelets (cor: 0.09), and negative regulation on lymphocytes (cor: -0.04). The results was showed in **Figure 2G**.

### Construction of cancer-related pain matrix

A total of 725 genes were obtained from GSE93157. A total of 12,098 targets were obtained from the GeneCards database. After the intersection of the two, a total of 662 cancer-related pain genes (CRPGs) were obtained. The expression levels of these 662 CRPGs were further extracted and the NSCLC Cancer-Related Pain Matrix was constructed.

### Consistency cluster analysis and differential expression analysis

**Figure 3:**
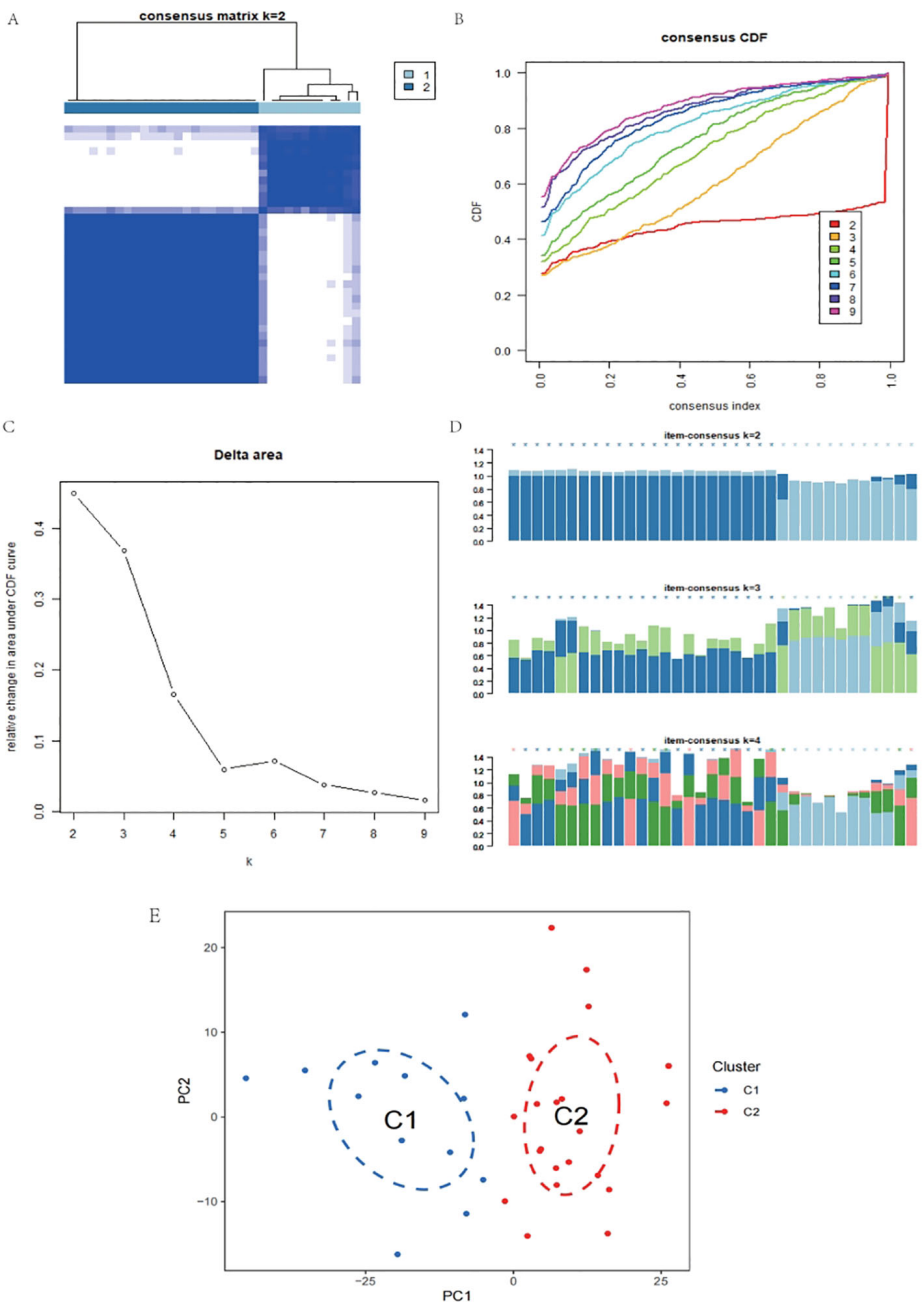
Consistency cluster analysis and principal component analysis. **(A)** When consensus matrix k=2, the samples were divided into two modules. **(B, C)** When consensus matrix k=2, the internal consistency performed best. **(D)** When consensus matrix k=2, consensus scores of all samples were displayed. **(E)** Principal component analysis indicated that when consensus matrix k=2, the samples can be well distinguished.

**Figure 4:**
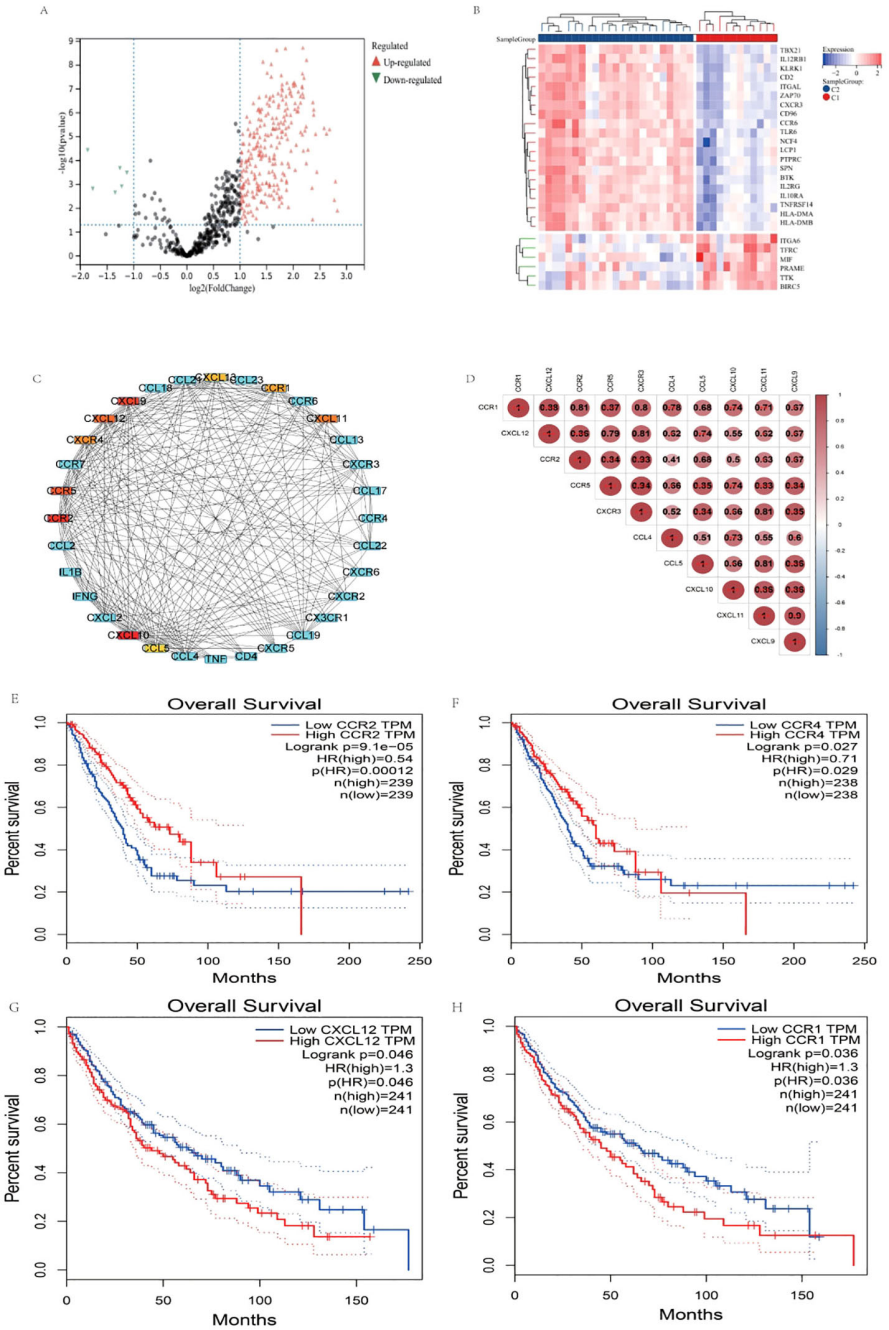
Differential expression analysis and survival analysis. **(A)** Volcanic map, red stands for up-regulation and green stands for down-regulation. **(B)** Heat map, red stands for up-regulation and blue stands for down-regulation. **(C)** MCC algorithm identified CXCL10, CCR2, CXCL9, CCR5, CXCL12, CXCL11, CXCR4, CCR1, CXCL13, and CCL5 hub 10 core targets. **(D)** Correlation analysis between 10 hub core targets. **(E)** OS of CCR2 in LUAD. **(F)** OS of CCR4 in LUAD. **(G)** OS of CXCL12 in LUSC. **(H)** OS of CCR1 in LUSC.

The K-means consistency clustering method was used for unsupervised learning, and the pain gene expression consistency within the NSCLC Cancer-Related Pain Matrix was divided into two modules, C2 and C1. There is a clear boundary between the two modules C1 and C2 **Figure 3A**. When consensus matrix k=2, the internal consistency performed best **Figures 3B, C**. Consensus scores of all samples were displayed **Figure 3D**. Principal component analysis indicated that when consensus matrix k=2, the samples can be well distinguished **Figure 3E**. Among them, 23 patients were classified into C2 and 12 patients were classified into C1 module. The limma package was used to perform differential expression analysis between C2 and C1, and a total of 420 differentially expressed genes (DEGs) were obtained. Among them, 398 were up-regulated and 22 were down-regulated **Figures 4A, B**.

### GO and KEGG functional enrichment analysis

GO and KEGG functional enrichment analysis of 420 DEGs showed that the interaction between cytokines and cytokine receptors is the main signaling pathway that mediates pain, **Supplementary Figure S2**, and these cytokines are mainly located on the cell membrane **Supplementary Figure S3**.

### PPI network construction and correlation analysis

Seventy-three targets involved in the interaction pathway between cytokines and cytokine receptors were imported into the string database, with Homo sapiens selected as the sample. The correlation coefficient was found to be 0.900. Subsequently, the MCC algorithm of the cytoHubba plug-in within the Cytoscape 3.8.2 software was utilized to identify the top 10 core targets. The analysis revealed that CXCL10, CCR2, CXCL9, CCR5, CXCL12, CXCL11, CXCR4, CCR1, CXCL13, and CCL5 were identified as the core top 10 targets **Figure 4C**. Correlation analysis between 10 hub targets indicated that there was a significant up-regulation relationship among the 10 factors **Figure 4D**.

### Survival analysis

To elucidate the potential association between 10 cytokines and survival in non-small cell lung cancer (NSCLC), a survival analysis was conducted utilizing data from the large-sample The Cancer Genome Atlas (TCGA) database, specifically focusing on the LUSC and LUAD subtypes. The findings revealed a significant correlation between high expression levels of CCR1 and CXCL12 in LUSC and poorer overall survival (OS) outcomes, **Figures 4G, H**, while in LUAD, high expression levels of CCR2 and CXCR4 were associated with improved OS **Figures 4E, F**. Additional corhorts from GSE14814, GSE73403 and GSE157010 indicated that high expression of CXCL12 showed potential significant difference correlation with poor prognosis of OS, and significant difference correlation with poor disease-free survival in GSE14814. **Supplementary Figure S4**.

### Tumor immune dysfunction and exclusion (TIDE) algorithm

**Figure 5:**
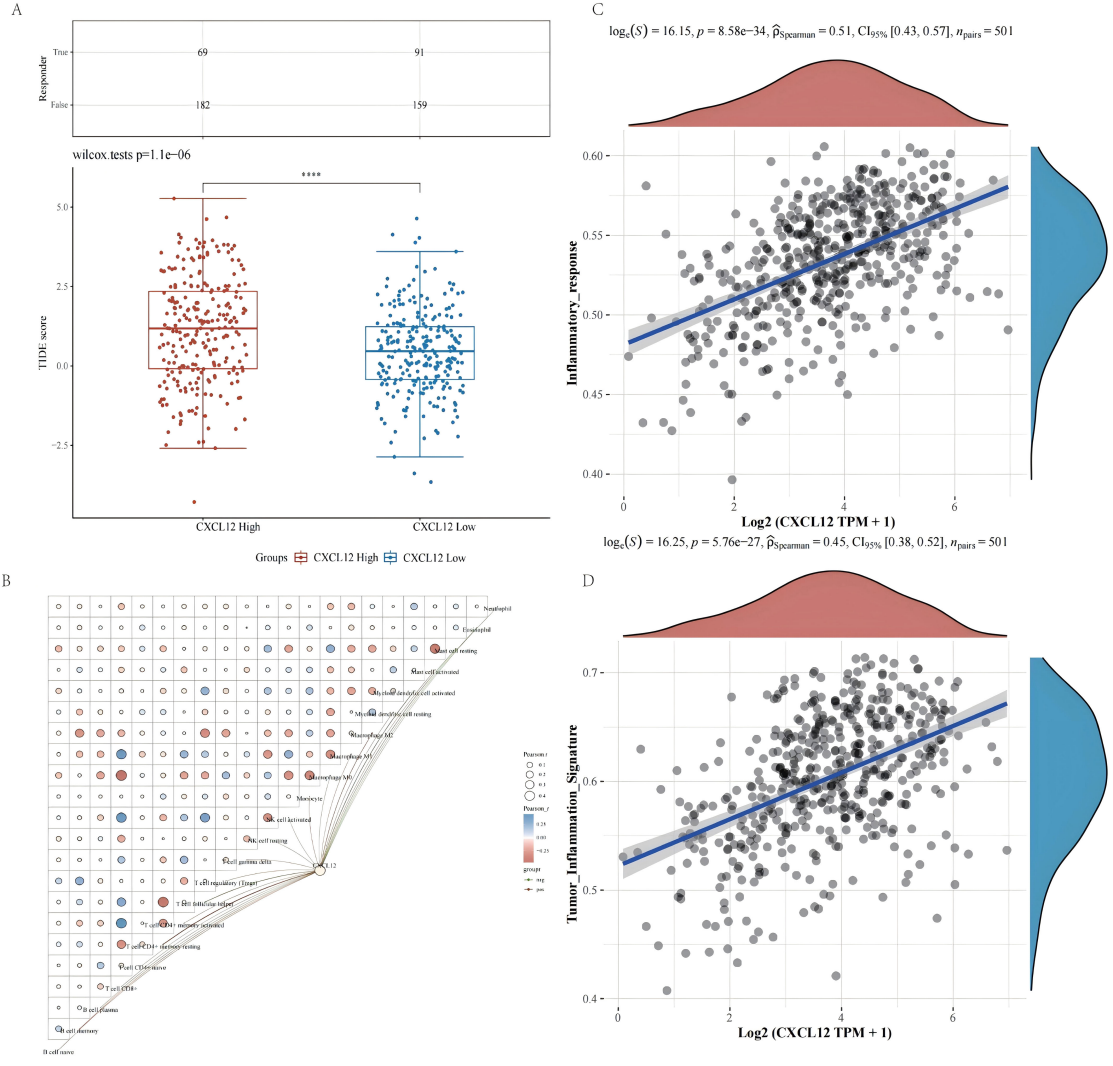
Efficacy evaluation of ICIs treatment and correlation analysis between CXCL12, immune cells, and inflammatory pathways. **(A)** Tumor immune dysfunction and exclusion algorithm indicated that high expression of CXCL12 was insensitivity to ICIs treatment. **(B)** CXCL12 can significantly up-regulate the expression of macrophages, monocyte cells and Tregs, and down-regulate CD8+ T cells and NK cells. **(C, D)** CXCL12 can significantly up-regulates inflammatory response pathway and tumor inflammation characteristic pathway.

To elucidate the impact of elevated levels of CCR1, CXCL12, CCR2, and CCR4 on the effectiveness of ICIs in the treatment of lung cancer, the TIDE algorithm was employed for predictive purposes. **Figure 5A**. The findings indicate that heightened CXCL12 expression is associated with diminished efficacy of immunotherapy in LUSC. Conversely, CCR1, CCR2, and CCR4 levels do not appear to influence the response to ICIs.

### Correlation analysis between survival related CRPGs, immune cells, and inflammatory pathways

In order to gain further insight into the potential effects of CXCL12 on immune cell populations, the CIBERSORT algorithm and Pearson correlation analysis were utilized. CXCL12 can significantly up-regulate the expression of macrophages, monocyte cells and T cell regulatory (Tregs), and down-regulate CD8+ T cells and NK cells **Figure 5B**. CXCL12 significantly up-regulates inflammatory response pathways and tumor inflammation characteristic pathways **Figures 5C, D**.

### ELISA assay

**Figure 6:**
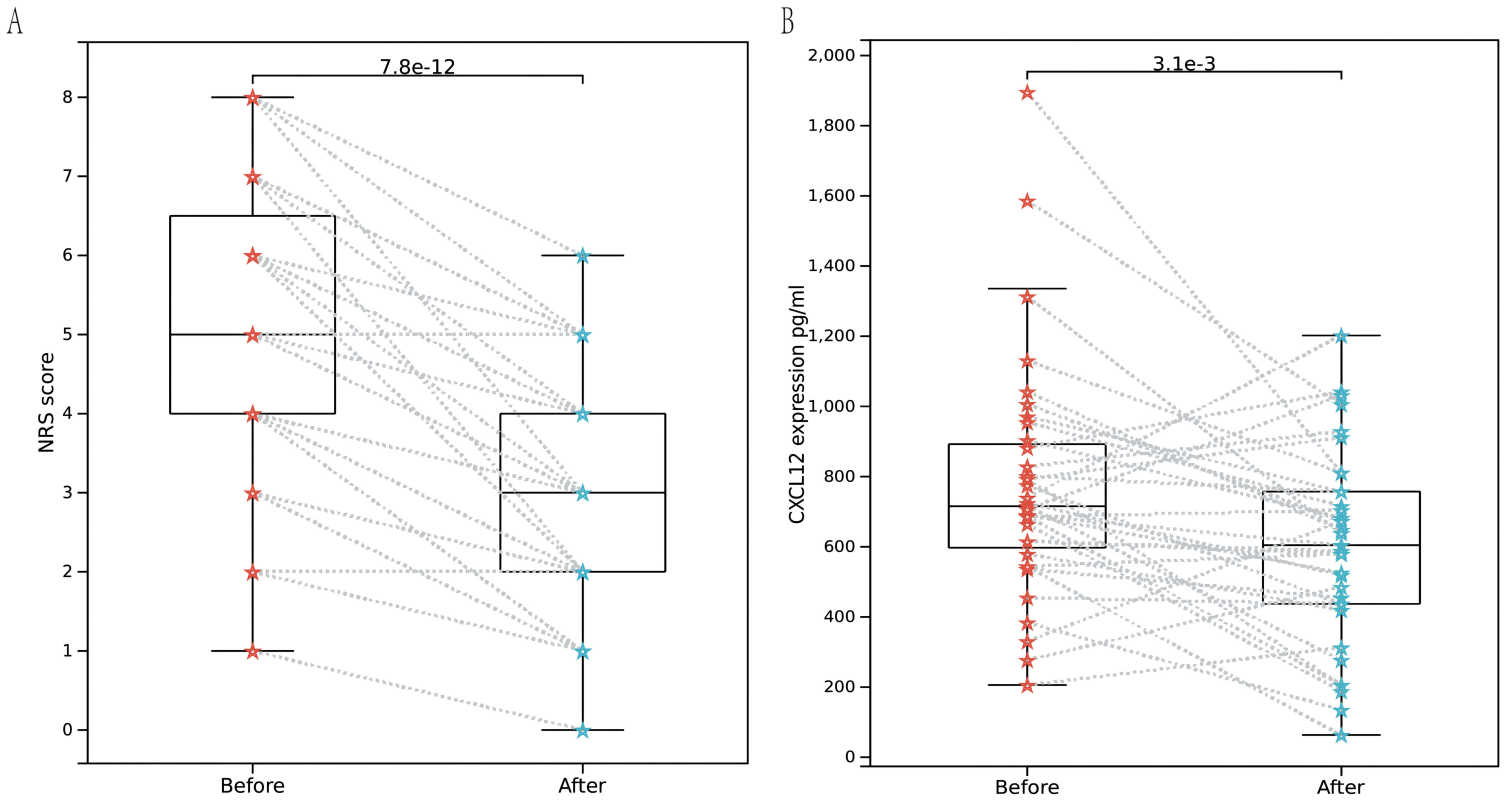
Enzyme-linked immunosorbent assay. **(A)** Rank sum test of NRS scores between baseline pain and pain relieved among 35 patients. **(B)** Rank sum test of NRS scores between baseline pain and pain relieved among 35 patients indicated that CXCL12 significantly decreased with p value of 3.1e-3.

35 patients with cancer pain were prospectively enrolled, all of whom had NRS scores recorded at baseline. Following cancer pain treatment, there was a significant decrease in NRS scores **Figure 6A**. ELISA detection revealed a significant decrease in serum CXCL12 levels after pain relief **Figure 6B**.

## Discussion

Pain is among the most frequently reported symptoms in individuals diagnosed with cancer (5). A recent systematic review, encompassing studies conducted between 2014 and 2021, determined the overall prevalence of pain in cancer patients to be 44.5%. Furthermore, 30.6% of these patients experienced moderate to severe pain (1). Cancer-related pain (CRP) is consistently linked to a diminished quality of life, attributable to psychological distress and impaired functioning (30). Several studies have suggested that inadequately managed pain may adversely affect survival rates in cancer patients (31, 32). The influence of pain on the effectiveness of immunotherapy remains a subject of debate in contemporary research. A retrospective study by Huan Zhou and colleagues indicated that baseline cancer pain serves as a negative prognostic indicator for lung cancer patients undergoing immunotherapy. Specifically, patients experiencing baseline cancer pain may exhibit poorer survival outcomes if they subsequently develop breakthrough pain (33).

CRP is hypothesized to be partially induced by tissue damage and inflammation within the tumor microenvironment (TME) through mechanisms that are not yet fully understood. Growing evidence indicates that the pathophysiology of chronic pain involves a complex interaction between the nervous and immune systems (34). Circulating immune cells, including neutrophils, monocytes, and T cells, are recruited to sites of tissue damage or inflammation and frequently infiltrate both the peripheral and central nervous systems (35, 36). The activation of these cells leads to the expression of a range of inflammatory mediators, such as cytokines, chemokines, lipids, and proteases. These mediators exert direct effects on peripheral sensory neurons and central second-order neurons, as well as indirect effects on other immune or local cells, thereby modulating pain.

Inflammation and cell-mediated immune function have been identified as factors associated with the efficacy of PD-1 blockade therapies (37, 38). Our study demonstrates that patients exhibiting elevated levels of the Platelet-Lymphocyte Ratio (PLR), Derived Neutrophil-Lymphocyte Ratio (dNLR), Systemic Inflammation Index (SII), and bone metastasis experience reduced efficacy of immunotherapy, both prior to and following Propensity Score Matching (PSM). The activation of neutrophils results in the upregulation of various proteins, including damage-associated molecular patterns (DAMPs), chemokines, and cytokines, such as vascular endothelial growth factor (VEGF), which contribute to enhanced tumor angiogenesis and the facilitation of distant metastasis (39). Furthermore, neutrophils can undergo degranulation, a process during which molecules such as defensins, myeloperoxidase, and lysozyme are secreted from intracellular granules into the extracellular environment, leading to tissue damage and promoting tumor metastasis (40, 41). Importantly, neutrophils can exhibit an immunosuppressive function in cancer, facilitating tumor progression primarily by inhibiting the recruitment of other immune cells to the tumor microenvironment (TME). Specifically, neutrophils are capable of releasing reactive oxygen species (ROS) (42), and enzymes such as arginase 1, which suppress the T-cell response within the TME. Notably, the secretion of interleukin-8 (IL-8) by cancer cells can stimulate neutrophils to release arginase into the TME (43). This enzyme degrades extracellular arginine, a crucial amino acid for T-cell activation and proliferation (44), thereby inhibiting the T-cell response.

In addition to the role of neutrophils, platelets typically exert a negative regulatory influence on immune checkpoint inhibitors (ICIs) therapy. Platelets facilitate the survival and proliferation of tumor cells through the secretion of various cytokines, including vascular endothelial growth factor (VEGF), transforming growth factor-β (TGF-β), and platelet-derived growth factor (PDGF) (45).Furthermore, chemokines associated with platelets have the capacity to modulate immune responses within the tumor microenvironment and influence tumor angiogenesis (46). Activated platelets are capable of engaging in both direct and mediated binding interactions with cancer cells. For instance, direct binding can occur between platelet P-selectin and cancer cell CD44. Additionally, fibrinogen can mediate binding between platelet GPIIb-IIIa and integrin αVβ3 on both cancer cells and cancer-associated angiogenic endothelial cells. Furthermore, von Willebrand factor can facilitate binding between platelet GPIbα and GPIbα-like motifs on cancer cells (47–51). These interactions enable activated platelets to effectively “cloak” cancer cells, thereby shielding them from immune surveillance within the circulatory system (49, 52, 53).

The effectiveness of immune checkpoint inhibitors (ICIs) is intricately linked to both the function and quantity of lymphocytes, particularly CD8+ T cells. These CD8+ T cells serve as the primary effector cells capable of infiltrating the tumor microenvironment of immunogenic tumors, thereby augmenting the therapeutic response to ICIs (54). Furthermore, various cytokines secreted by lymphocytes, including interferon-gamma (IFN-γ) and tumor necrosis factor-alpha (TNF-α), contribute to tumor suppression and extend the survival of cancer patients (55).

In addition to the involvement of peripheral blood inflammatory cells, our findings indicate that bone metastases significantly contribute to the limited efficacy of immune checkpoint inhibitors (ICIs) treatment. The skeletal system is frequently affected during metastatic progression, resulting in bone-related complications such as severe pain, pathological fractures, and hypercalcemia, all of which substantially diminish patients’ quality of life (56). Furthermore, the bone microenvironment is characterized by a distinct immunosuppressive milieu (57). Notably, bone marrow-derived suppressor cells (MDSCs) have been implicated in the suboptimal therapeutic outcomes associated with ICIs (58). MDSCs have the capacity to impede the anti-tumor activities of CD8+ T cells and NK cells, thereby exerting a detrimental influence on immune regulation.

Although it was clear in retrospective analysis that pain will lead to poor curative effect of ICIs, the key molecules and mechanisms that played a role were still unclear. We found that CXCL12 played an important role in this process. Through GEO data set, we identified CXCL12, a pain-related core target in patients receiving ICIs treatment. Studies have shown that the activation of CXCL12/CXCR4 signaling pathway can up-regulate the phosphorylation of extracellular signal regulated kinase (ERK) in spinal cord or the phosphorylation and expression level of sodium channel Nav1.8 in DRG, and activate neurons to generate excitement, thus causing chronic pain (59, 60). However, the specific inhibition of CXCL12/CXCR4 signal pathway or the expression of its upstream and downstream channels can inhibit pain sensitization. The activation of CXCL12/CXCR4 axis also plays an important role in the formation of cancer pain. The strong interaction between signal transducer and activator of transcription 3 (STAT3) and p300 leads to the high expression of CXCL12 in dorsal horn neurons, which leads to neuropathic pain induced by anti-tubulin chemotherapy drugs (such as paclitaxel) (61). CXCL12/CXCR4 signaling pathway activates sensitized neurons, astrocytes and microglia through mitogen-activated protein kinase (MAPK), promotes the release of inflammatory factors, such as IL(interleukin) and TNF, and causes persistent bone cancer pain (62).

Further using TCGA data set, it was found that the prognosis of patients with high expression of CXCL12 in LUSC was significantly worse than that of patients with low expression. CXCL12 promotes tumor angiogenesis by targeting vascular endothelial cells and cooperating with vascular endothelial growth factor (VEGF) (63), and also promotes tumor cell proliferation and survival (64, 65). In addition, CXCL12-CXCR4 signaling pathway is involved in the invasion and metastasis of cancer cells. In many retrospective studies, it was found that CXCR4 is the most widely expressed chemokine receptor in tumor cells, which is responsible for the metastasis of tumor cells to lung, liver and bone marrow, which are the most common metastasis destinations in many cancers (66). On the other hand, CXCL12 has immunosuppressive effect, which will reduce the efficacy of ICIs. CXCL 12/CXCR 4 axis can regulate the recruitment of specific immune cells in TME, and drive the immune cells expressing CXCR 4 to polarize toward immunosuppression phenotype. Previous evidence shows that CXCL12 mediates plasma cell-like DC transport to tumor and Treg cells homing to bone marrow microenvironment (67); In addition, it stimulates antigen-specific T lymphocytes and macrophages to express pro-angiogenic factors by mediating the polarization of T cells to Treg (68, 69) and producing DC with poor function (70).

In melanoma mouse model, high levels of CXCL12 can repel T effector cells expressing CXCR4 (71), limiting their infiltration and killing of tumor cells (72, 73). In clinical specimens, cancer cells in breast, colorectal, and breast cancer appear to be surrounded by CXCL12-KRT19 heterodimers, potentially making them resistant to immunotherapy (74). A study found that T cells’ movement may be affected by CXCL12 dimer inhibiting F-actin polymerization (75). Tumor-related lymphatic vessels control CD8+ T cell migration through CXCL12, and accumulating antigen-specific CD8+ T cells in tumors was crucial for effective immunotherapy (76).

## Conclusion

Baseline pain was identified as an independent prognostic risk factor for the diminished efficacy of ICIs in the treatment of NSCLC. Baseline pain has the potential to inhibit the tumor immune micro-environment by increasing the presence of inflammatory cells in the peripheral blood cell counts, specifically neutrophils and monocytes, ultimately leading to reduced responsiveness to ICIs. The chemokine CXCL12 was implicated in both pain modulation and immune regulation, with its up-regulation further contributing to the presence of inflammatory cells in the peripheral blood cell counts, such as monocytes and macrophages, while simultaneously suppressing the activity of NK cells and CD8+ T cells. This phenomenon results in suboptimal therapeutic outcomes with ICIs, particularly in patients with LUSC. However, this study also has some limitations. First, the conclusions based on retrospective analysis were weak in terms of evidence level. Secondly, there are many variables included in this study, but the sample size is relatively insufficient.

## Data Availability

The original contributions presented in the study are included in the article/**Supplementary Material**. Further inquiries can be directed to the corresponding authors.

## Supplementary material

Supplementary Figure 1 Differential analysis of Risk scores. **(A)** Bone metastasis before PSM. **(B)** Pain before PSM. **(E)** Bone metastasis after PSM. **(D)** Pain after PSM. **(E)** Age after PSM. **(F)** dNLR after PSM.

Supplementary Figure 2 KEGG functional enrichment analysis of 420 DEGs showed that the interaction between cytokines and cytokine receptors was the main signaling pathway that mediates pain.

Supplementary Figure 3 GO functional enrichment analysis of 420 DEGs showed that these cytokines were mainly located on the cell membrane. They were closely related to biological processes like immune system processes and molecular functions like signaling receptor activity.

Supplementary Figure 4 Survival analysis of CXCL12 in external cohorts. **(A)** OS in GSE14814. **(B)** DFS in GSE14814. **(C)** OS in GSE73403. **(D)** OS in 157010.
