# Supplementary figures and images for: Unraveling impact and potential mechanisms of baseline pain on efficacy of immunotherapy in lung cancer patients: a retrospective and bioinformatic analysis
Source: Front Immunol. 2024 Nov 25;15:1456150. doi: 10.3389/fimmu.2024.1456150 (PMC11625792; doi:10.3389/fimmu.2024.1456150)

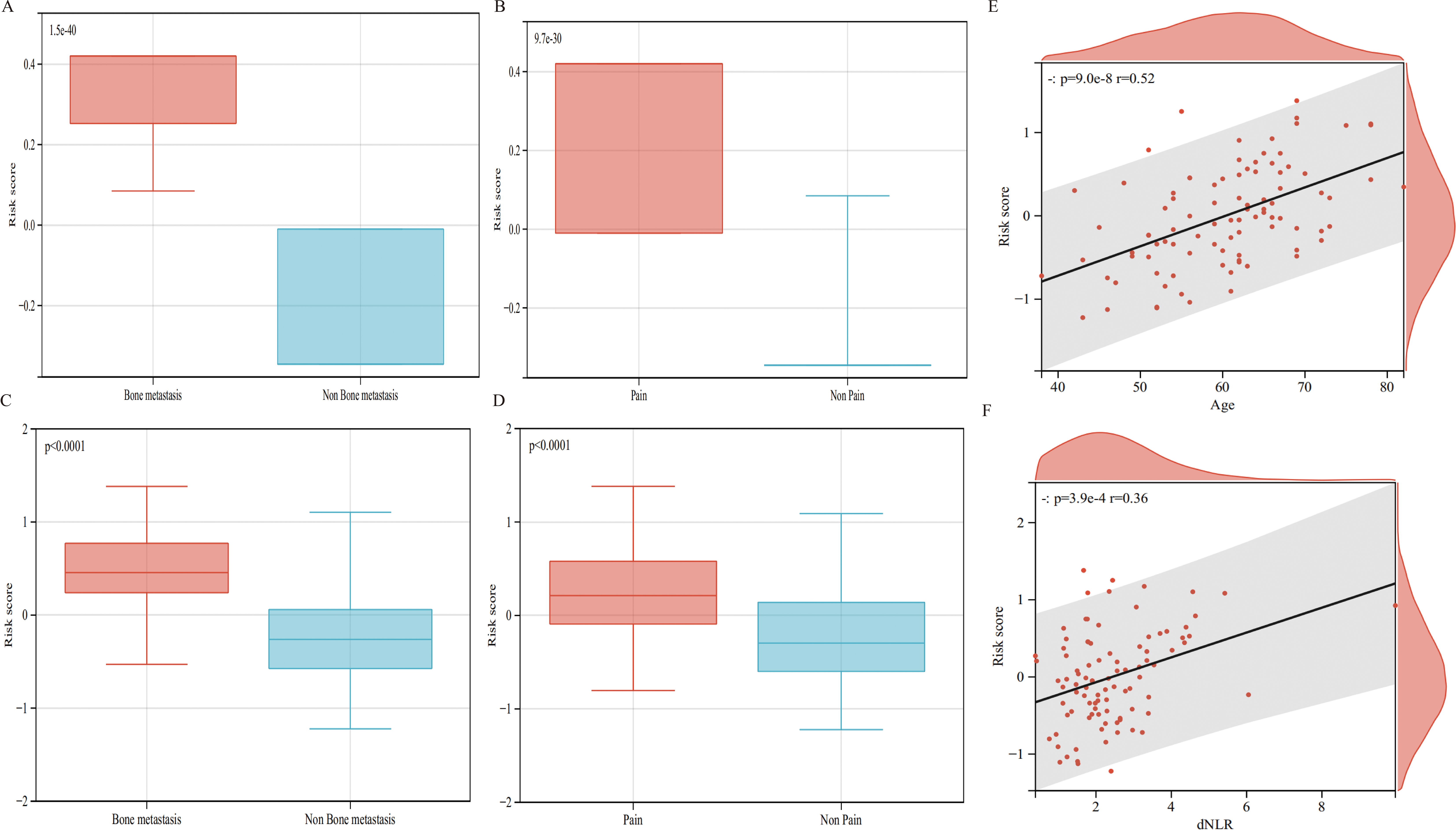

Supplement: Supplementary Figure 1 — Differential analysis of Risk scores. (A) Bone metastasis before PSM. (B) Pain before PSM. (E) Bone metastasis after PSM. (D) Pain after PSM. (E) Age after PSM. (F) dNLR after PSM. [file Image1.jpg]

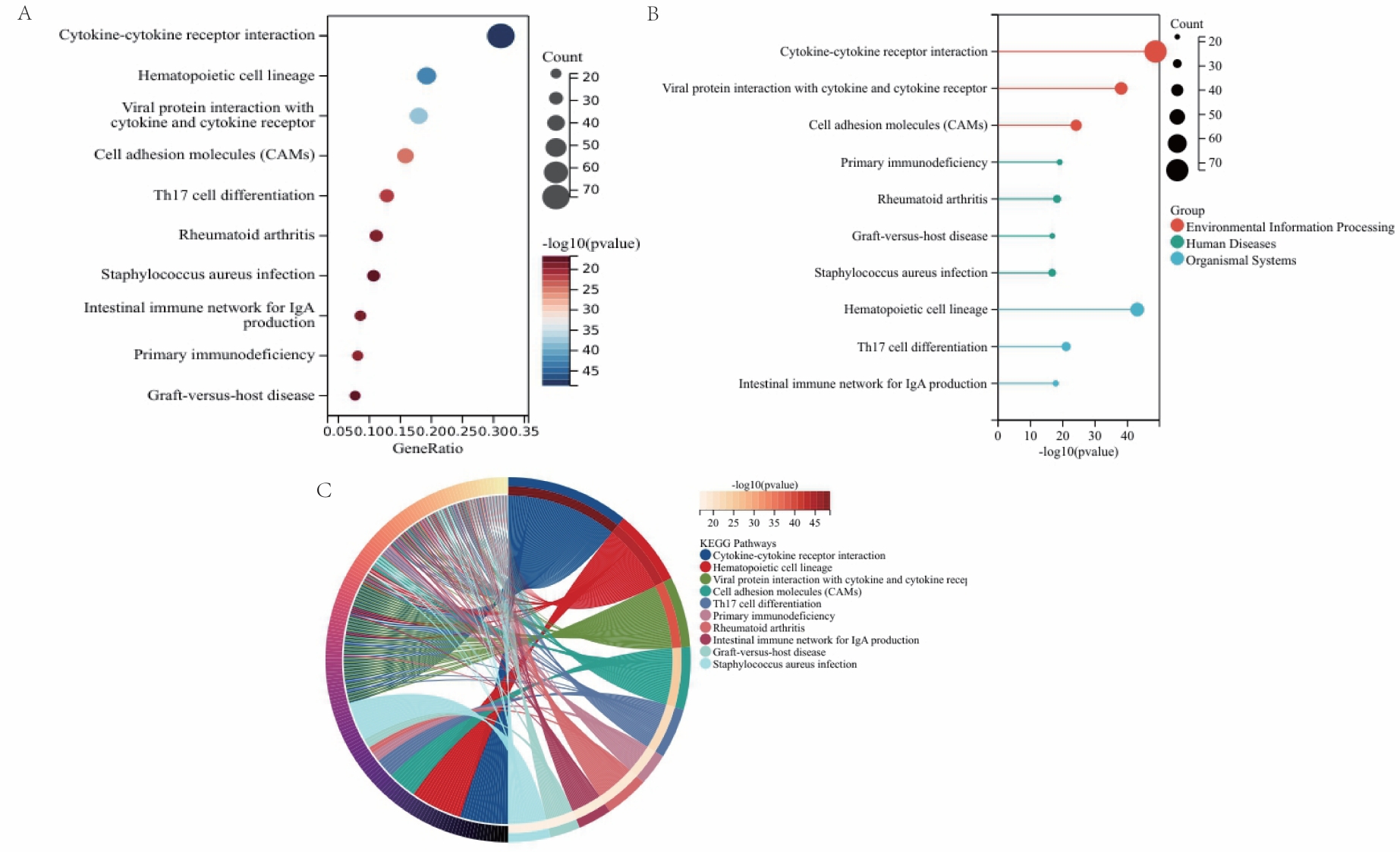

Supplement: Supplementary Figure 2 — KEGG functional enrichment analysis of 420 DEGs showed that the interaction between cytokines and cytokine receptors was the main signaling pathway that mediates pain. [file Image2.jpg]

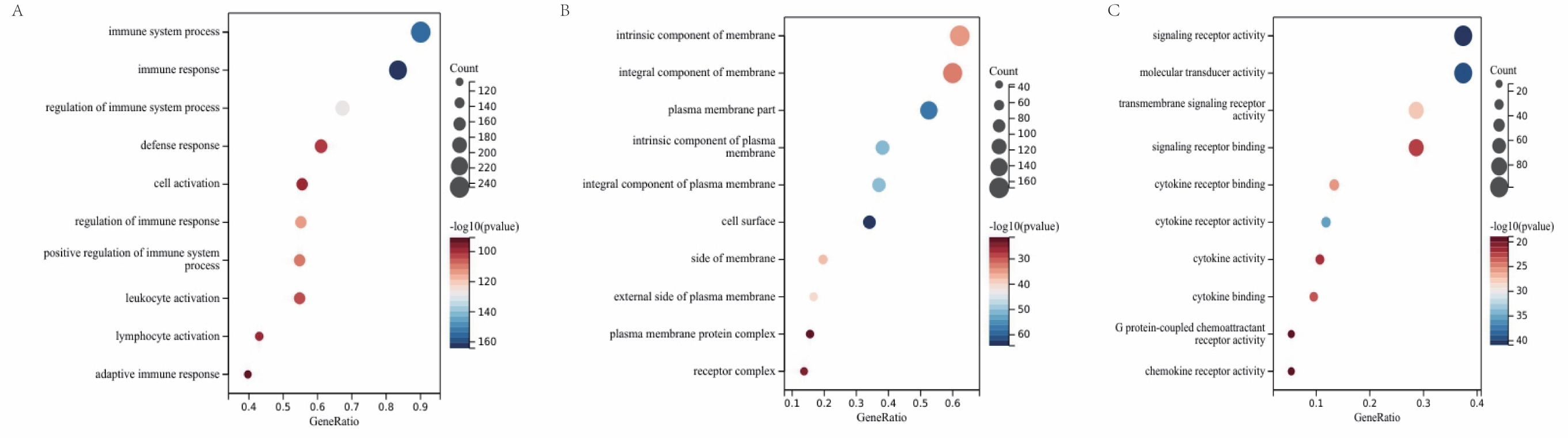

Supplement: Supplementary Figure 3 — GO functional enrichment analysis of 420 DEGs showed that these cytokines were mainly located on the cell membrane. They were closely related to biological processes like immune system processes and molecular functions like signaling receptor activity. [file Image3.jpg]

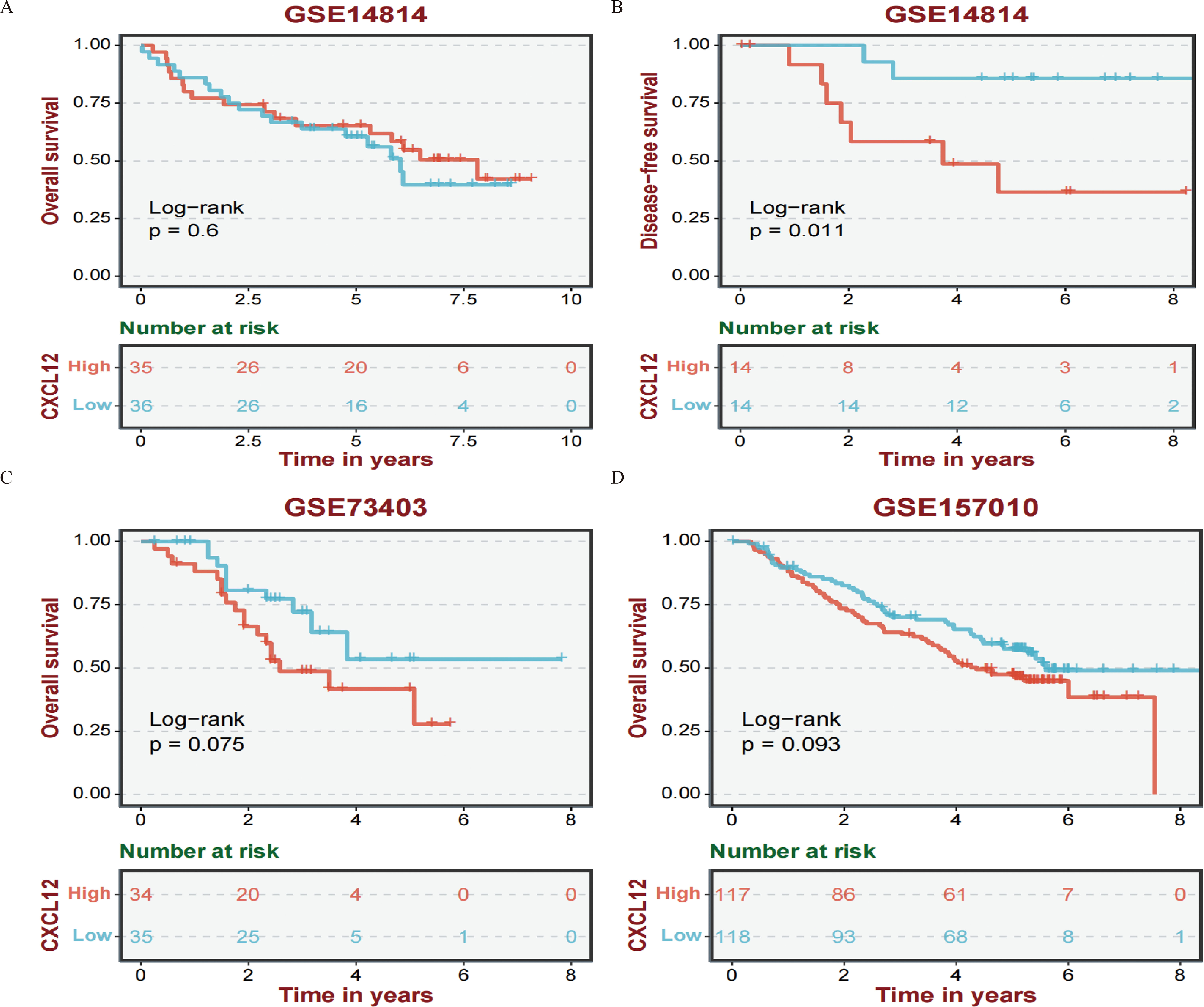

Supplement: Supplementary Figure 4 — Survival analysis of CXCL12 in external cohorts. (A) OS in GSE14814. (B) DFS in GSE14814. (C) OS in GSE73403. (D) OS in 157010. [file Image4.jpg]
